# Transmedulla Neurons in the Sky Compass Network of the Honeybee (*Apis mellifera*) Are a Possible Site of Circadian Input

**DOI:** 10.1371/journal.pone.0143244

**Published:** 2015-12-02

**Authors:** Maximilian Zeller, Martina Held, Julia Bender, Annuska Berz, Tanja Heinloth, Timm Hellfritz, Keram Pfeiffer

**Affiliations:** Department of Biology - Animal Physiology, Philipps-University Marburg, Marburg, Germany; Lund University, SWEDEN

## Abstract

Honeybees are known for their ability to use the sun’s azimuth and the sky’s polarization pattern for spatial orientation. Sky compass orientation in bees has been extensively studied at the behavioral level but our knowledge about the underlying neuronal systems and mechanisms is very limited. Electrophysiological studies in other insect species suggest that neurons of the sky compass system integrate information about the polarization pattern of the sky, its chromatic gradient, and the azimuth of the sun. In order to obtain a stable directional signal throughout the day, circadian changes between the sky polarization pattern and the solar azimuth must be compensated. Likewise, the system must be modulated in a context specific way to compensate for changes in intensity, polarization and chromatic properties of light caused by clouds, vegetation and landscape. The goal of this study was to identify neurons of the sky compass pathway in the honeybee brain and to find potential sites of circadian and neuromodulatory input into this pathway. To this end we first traced the sky compass pathway from the polarization-sensitive dorsal rim area of the compound eye via the medulla and the anterior optic tubercle to the lateral complex using dye injections. Neurons forming this pathway strongly resembled neurons of the sky compass pathway in other insect species. Next we combined tracer injections with immunocytochemistry against the circadian neuropeptide pigment dispersing factor and the neuromodulators serotonin, and γ-aminobutyric acid. We identified neurons, connecting the dorsal rim area of the medulla to the anterior optic tubercle, as a possible site of neuromodulation and interaction with the circadian system. These neurons have conspicuous spines in close proximity to pigment dispersing factor-, serotonin-, and GABA-immunoreactive neurons. Our data therefore show for the first time a potential interaction site between the sky compass pathway and the circadian clock.

## Introduction

Honeybees possess a time-compensated sun-compass, which enables them to use the solar azimuth, i.e. the horizontal component of the sun’s position, as a reference direction for navigation [1]. In his seminal studies, Karl von Frisch was able to show that bees can infer the solar azimuth from a patch of blue sky [2]. Using a polarizer von Frisch manipulated the bee’s orientation during waggle dances on a horizontal comb, showing for the first time polarization-sensitivity and its use for orientation in any animal [3]. Employing polarized light as a navigational cue in lieu of the sun is possible, because the scattering of sunlight in the atmosphere leads to a regular pattern of electric field vectors (*E*-vectors) that are oriented tangentially to concentric circles around the sun [4]. In addition, scattering leads to a chromatic and an intensity gradient across the sky [5, 6], both of which can be the source of directional information for orienting insects [7, 8]. Recent evidence from desert ants of the genus *Cataglyphis* shows that these animals can learn homing directions using only the sun as an orientational cue and later use this information to navigate solely by the polarization pattern and vice versa [9]. This suggests that the neuronal correlates of the sun-compass and polarization compass can either exchange information or that they are identical. The latter hypothesis is supported by the finding that the same neurons in the anterior optic tubercle of the locust that code for *E*-vector orientation of polarized light also code for the azimuthal position and wavelength of unpolarized light stimuli [10, 11]. Similarly, polarization-sensitive neurons in monarch butterflies and dung beetles also code for the azimuth of an unpolarized light stimulus, albeit wavelength sensitivity in these species is either lacking (monarch) or has not been tested (dung beetle) [12, 13]. Most insects, including honeybees, perceive the *E*-vector of polarized light with a specialized dorsal rim area (DRA) of their compound eye [14]. As polarization-vision systems are normally homochromatic, perception of the color gradient and the direct sun light is performed with the remainder of the compound eye [10, 14]. It is currently not clear at which stage of the sky compass pathway these pieces of information are integrated, but electrophysiological data from locusts suggest that a central layer of the medulla might be important for this task [15].

In order for an animal to use the sun as a spatial reference cue throughout the entire day, it has to continuously update its orientation with respect to the changing solar azimuth. An intuitive demonstration of this time-compensation capability was provided by Martin Lindauer [16] who observed that bees that performed waggle dances for extended periods of time continuously updated their dancing directions to match the changing solar azimuth. In addition to compensating for changes in solar azimuth, a system that integrates polarized and unpolarized celestial cues also needs to compensate for potential cue conflicts that arise from changes in solar elevation. Such a compensation mechanism has been shown to be present in neurons of the anterior optic tubercle, but the underlying mechanisms are unknown [11]. A fundamental requirement for both types of time-compensation is that neurons representing solar azimuth receive time information from neurons of the circadian clock.

In the cockroach *(Rhyparobia maderae)* and the fruit fly *(Drosophila melanogaster)*, some neurons of the accessory medulla that contain the neuropeptide pigment dispersing factor (PDF), have been shown to be the pacemakers of the circadian clock. [17–19]. PDF is an output signal of the insect circadian clock [20–22]. In honeybees the expression level of pdf mRNA shows a circadian rhythm both under light-dark and constant darkness conditions [23]. Both PDF and the biogenic amine serotonin (5-hydroxytrypamine, 5HT) have been shown to mediate circadian effects in the visual system of insects, including size changes of lamina monopolar neurons in the housefly [24], as well as sensitivity changes in visual interneurons of crickets [25–27] and ERGs of blowflies [28]. In honeybees, 5HT has been shown to modulate the sensitivity of visual interneurons in the lobula [29].

There is now a large body of work regarding the navigational capabilities of honeybees with respect to celestial cues, but beyond the level of the polarization sensitive photoreceptors of the DRA [30], neither the neural substrates nor mechanisms that underlie this sophisticated behavior are known. In other insect species the morphology and physiology of polarization-sensitive neurons in the brain have been studied in some detail, but a locus for time-compensation has not yet been identified. The goals of this study were to morphologically characterize the honeybee sky compass pathway and to identify potential sites of interaction with the circadian clock within this pathway.

## Methods

### Animals

Honeybees *(Apis mellifera)* were obtained from hives maintained at the University of Marburg, Germany. Between April and October, bees were kept outdoors. In October, hives were moved to a greenhouse under natural light/dark conditions, temperatures between 20 and 25°C, and relative humidities between 60% and 80%. Bees could freely forage for ground pollen, honey water (20–30% v/v), and water within a volume of 2 m x 2 m x 2 m. All experiments were performed on foraging worker honey bees collected outside the hive. According to the German animal welfare act, no approval is required for experiments on insects.

### Preparation 1

Animals were cold anesthetized on ice or in the refrigerator. To immobilize the animals they were attached to a custom made holder using dental wax. The head capsule was opened frontally and trachea, air sacs and glands were removed to expose the brain.

### Mass dye injections

To trace the sky compass pathway, we used mass injection of dextrans that were either coupled to fluorescent dyes (dextran Texas Red, 3000 MW, lysine fixable; dextran Alexa Fluor 488, 10000 MW, anionic, fixable) or to biotin (3000 MW, lysine fixable, Molecular Probes; all dextrans: Molecular Probes, Eugene, USA). To stain photoreceptors of the dorsal rim area, the cornea and crystalline cone layer of the DRA were removed using a microscalpel. A tracer crystal was placed into the opening, which was then sealed with petroleum jelly to avoid desiccation. For injection into the anterior optic tubercle and the dorsal rim area of the medulla, intracellular recording pipettes were pulled from borosilicate glass (inner diameter 0.75 mm, outer diameter 1.5 mm, Hilgenberg, Malsfeld, Germany) using a p-97 horizontal puller (Sutter Instrument, Novato, CA, USA) and broken to a tip diameter of approximately 5–30 μm. The tip of the pipette was dipped into petroleum jelly which allowed us to pick up a small tracer crystal. After removing the neural sheath above the target area, the handheld pipette was inserted into the brain to deposit the dye. Superficial excess dye was removed by extensive rinsing with honey bee Ringer solution (in mM: NaCl 130, KCl 5, MgCl_2_ 4, HEPES 15, Glucose 25, Sucrose 160). After the injection, the previously removed piece of cuticle from the head capsule was replaced and covered with a tissue soaked in bee Ringer. The animals were placed overnight in a moist chamber at 4°C to allow for tracer uptake and diffusion.

### Extracellular iontophoretical dye injections

To stain small numbers of neurons (1–20) with processes in the anterior optic tubercle (AOTU), we used extracellular iontophoretical dye injections. Intracellular recording pipettes with resistances between 100 and 300 MΩ in the tissue were fabricated as described above. Electrode tips were filled with 4% Neurobiotin (Vector Laboratories Burlingame, USA) in 1 M KCl and backed with 1–2.5 M KCl. After removing the neural sheath, electrodes were frontally inserted into the AOTU using a micromanipulator (Leica Microsystems, Wetzlar, Germany). To eject the tracer, and to create an electroporating electrical field [31], we applied rectangular current pulses of 10 nA amplitude with a frequency of 1 Hz and a duty cycle of 50% for 15 to 45 minutes, using a custom built amplifier.

### Preparation 2

Brains were dissected from the head capsule during continuous submersion in fixative solution. Brains were then fixated overnight at 4°C. In some preparations, the entire head capsule was fixated overnight and the brain was removed the next day. Fixative solutions depended on the type of subsequent antibody staining and are listed in Table 1. Specimen that were not immunostained, were fixated in 4% paraformaldehyde, 0.2% glutaraldehyde, and 0.2% saturated picric acid in 0.1 M phosphate buffered saline (PBS, pH 7.4).

**Table 1:** Primary antibodies, dilutions and fixatives.

Antibody	Raised against	Raised in	Fixative	Working dilution	Source	reference
Anti-synapsin	*Drosophila* SYNORF1-GSA fusion protein	mouse	4% PFA, 0.2% saturated PA, 0.25% GA	1:50	Dr. E. Buchner, Würzburg, Germany	Klagges et al. 1996, RRID: AB_2315425
Anti-*Apis* pigment dispersing factor	*Apis* PDF coupled to KLH with MBS	rabbit	4% PFA	1:2000	Dr. M. Shimohigashi, Fukuoka, Japan	Sumiyoshi et al. 2011
Anti *Uca pugilator* pigment dispersing hormone	conjugate of synthetic *Uca pugilator* β-PDH and bovine thyroglobulin	rabbit	4% PFA, 7.5% saturated PA	1:1000	Dr. H. Dircksen, Stockholm, Sweden	Dircksen et al. 1987, RRID:AB_2315088
Anti-5HT	5HT coupled to BSA with PFA	rabbit	4% PFA, 7.5% saturated PA	1:1000	ImmunoStar, Cat. No. 20080	ImmunoStar; histochemical 5HT antisera specification sheet, RRID:AB_572263
Anti-GABA No. 9/24;	GABA coupled to KLH with GA	rabbit	4% PFA, 0.5% GA	1:500	Dr. T. Kingan	Hoskins et al. 1986, RRID:AB_2314457

GA, glutaraldehyde; GSA, glutathione-S-transferase; KLH, keyhole limpet hemocyanin; MBS, m-maleimidobenzoyl-N-hydroxysuccinimide ester; PA, picric acid; PFA, paraformaldehyde.

### Biotin/streptavidin labeling

In preparations where either Neurobiotin or biotinylated streptavidin was injected, neurons were visualized through incubation of the tissue with streptavidin conjugated to Cy3 (1:1000, Jackson Immunoresearch, West-Grove, PA, USA, RRID: AB_2337244). Streptavidin was applied for 3 to 5 days in 0.1 M PBS, 0.3% TrX, and 0.02% sodium azide. In brains that underwent subsequent immunostaining, streptavidin was added to either the primary and secondary, or only the secondary antibody solution.

### Antibody characterization

For immunolabeling, we used polyclonal antibodies against *Apis mellifera* pigment dispersing factor (NSELINSLLGLPKNMNNA-NH2, PDF), *Uca pugilator* β-pigment dispersing hormone (NSELINSILGLPKVMNDA-NH2, PDH), γ-aminobutyric acid (GABA), and serotonin (5-hydroxytryptamine, 5HT) and a monoclonal antibody against the synaptic vesicle protein synapsin (Table 1).

The polyclonal PDF antiserum (kindly provided by Dr. M. Shimohigashi, Fukuoka University, Japan) was raised in rabbits against synthetic Cys-attached *Apis mellifera* PDF, which was conjugated to keyhole limpet hemocyanin (KLH). Specificity of the antibody was tested using ELISA. No cross reaction was found to the PDFs of *Bombyx mori* or *Gryllus bimaculatus*, but about 20% cross reactivity was observed for the PDF of *Musca domestica*. In-situ hybridization, using an antisense cRNA probe that hybridized specifically to all types of pdf mRNA, labeled the same number of cell bodies (n = 14) in the same region as the polyclonal antibody did [23].

The polyclonal PDH antiserum (kindly provided by Dr. Heinrich Dircksen, Stockholm University, Sweden, RRID:AB_2315088) was raised in rabbits against a glutaraldehyde conjugate of synthetic *Uca pugilator* pigment dispersing hormone and thyroglobulin [32]. This antiserum has been well characterized through ELISAs and immunoassays [32, 33]. It has been previously used to stain PDH-ir neurons in a large number of different insect species including the honeybee [17, 34–36, 36–39]. Staining patterns in the honeybee brain using either the PDF antiserum or the PDH antiserum are highly similar, with a slightly larger number of cell bodies stained by the PDH antibody [23, 37].

The polyclonal antiserum against 5HT was purchased from ImmunoStar (Hudson, NY, USA, Cat# 20080, RRID:AB_572263). It was raised in rabbit against 5HT conjugated to bovine serum albumin. According to the manufacturer’s datasheet this antiserum exhibited no cross reactivity to 5-hydroxytryptophan, 5-hydroxyindole-3-acetic acid, or dopamine in Bn-SA/HRP labeling assays. Preadsorption of the antiserum with 5HT-BSA conjugate abolished, or strongly reduced immunoreactivity in bumblebee brains [40], while preadsorption with BSA had no effect on the staining [41]. The staining pattern in the optic lobe using this antiserum was virtually identical to that described by Ehmer and Gronenberg [42], who used a different antiserum (DiaSorin, Stillwater, MN).

The antiserum against GABA (kindly provided by Dr. T. G. Kingan, No. 9/24, RRID:AB_2314457) was raised in rabbit against GABA conjugated to KLH and has been affinity purified against KLH [43]. Preadsorption experiments on brain sections of *Manduca sexta*, and *Schistocerca gregaria* have previously demonstrated the specificity of the antiserum [43, 44].

The monoclonal antibody against the synaptic vesicle protein synapsin (kindly provided by Dr. E. Buchner Würzburg, Germany, SYNORF1, RRID: AB_2315425) was raised against fusion proteins of glutathione-S-transferase and *Drosophila* SYN1 protein [45] and has been used in many insect species, including honeybees, to label synaptic neuropils [45–49]. The specificity of the antibody has been characterized by Klagges et al. [45]. *Drosophila* synapsin null mutants (syn79) completely lack immunoreactivity [50].

### *Apis* PDF immunohistochemistry on whole mount brains

Brains were washed in PBS with 0.3% Triton X-100 (TrX, Sigma, Deisenhofen, Germany) and incubated overnight at 4°C in blocking solution containing 5% normal goat serum (NGS, Dianova, Hamburg, Germany), 0.3% TrX, and 0.02% sodium azide in PBS. After rinsing in PBS, brains were incubated for five days at 4°C with the primary antibody solution containing anti-PDF antiserum at a dilution of 1:1000, 1% NGS, 0.5% TrX and 0.02% sodium azide in PBS. After washing in PBS, brains were incubated for three days at 4°C with the secondary antibody solution. It contained goat anti-rabbit IgG conjugated to Cy2 (1:300, Dianova, Hamburg, Germany), 1% NGS and 0.5% TrX and 0.02% sodium azide in PBS. After several rinses in PBS, brains were dehydrated in an ascending ethanol series (25%, 50%, 70%, 90%, 95%, 100%, 15 min each) and transferred to a 1:1 mixture of 100% ethanol and methylsalicylate for 30 min. Eventually they were cleared in methylsalicylate for at least 45 min and mounted between two coverslips using Permount (Fisher Scientific, Pittsburgh, PA, USA). To avoid deformation of the brain, eight stacked hole reinforcements (Zweckform, Oberlaindern, Germany) were used as a spacer.

### PDH immunostaining on gelatin sections from rehydrated brains

Brains were washed, dehydrated, cleared and mounted as described in the previous section. Tracer injection was evaluated using a fluorescence microscope (Zeiss Axioskop, Zeiss, Jena Germany) and brains with successful injections into the AOTU were selected for further processing. To recover the embedded brains, Permount was removed by incubation in xylene for 1–3 hours at room temperature. Following a descending ethanol series (100%, 95%, 90%, 70%, 50%, 25%) brains were washed in PBS, embedded in albumin/gelatin (12% ovalbumin, 4.8% gelatin in demineralized water) and fixated overnight at 4°C with 8% formaldehyde in PBS. Brains were sectioned at 130 μm in the frontal plane using a vibrating blade microtome (VT1200 S, Leica Microsystems, Wetzlar, Germany). After several washes in 0.01 M PBS with 0.3% TrX, sections were pre-incubated with 5% NGS, 0.02% sodium azide and 0.01 M PBS with 0.3% TrX overnight at 4°C. The primary antibody solution was then applied for 5 days at 4°C. It contained PDH antiserum (1:1000), 1% NGS, 0.3% TrX, 0.02% sodium azide and 0.01 M PBS. After extensive rinses in 0.01 M PBS with 0.3% TrX, the secondary antibody solution was applied for 3 days at 4°C. It consisted of goat anti rabbit IgG conjugated to Cy2 (1:200, Dianova, Hamburg, Germany), 1% NGS, 0.3% TrX, 0.02% sodium azide and 0.01 M PBS. After washing, dehydrating, and clearing, as described for the whole mount preparations, sections were mounted on microscopic slides using Permount and spacers.

### GABA and 5HT immunohistochemistry on gelatin sections

Brains were embedded in albumin/gelatin and fixated overnight at 4°C with 8% formaldehyde in PBS and sliced in the frontal plane at a thickness of 40 μm using a vibrating-blade microtome (VT1200 S, Leica Microsystems, Wetzlar, Germany).

For GABA immunohistochemistry, slices were washed with saline substituted Tris-buffer (SST; pH 7.4) containing 0.1% TrX. To reduce background fluorescence caused by Schiff’s bases as a result from glutaraldehyde fixation, free floating sections were treated for 10 minutes with 10 mg/ml NaBH_4_ and 0.1% TrX in 0.01 M phosphate buffer [51]. After rinsing with 0.1% TrX in SST, sections were pre-incubated for one hour at room temperature with 10% normal donkey serum (NDS; Dianova, Hamburg, Germany), 0.5% TrX and SST. The primary antiserum against GABA was diluted 1:500 in a solution of 1% NDS, 0.02% sodium azide and 0.5% TrX in SST. Slices were incubated overnight at 30°C in an incubator. After washing in SST containing 0.1% TrX, the secondary antibody solution, which was composed of donkey anti-rabbit IgG Cy2 (1:200; Dianova, Hamburg, Germany) 1% NDS and 0.5% TrX in SST, was applied for one hour at room temperature. After washing with 0.1% TrX in SST sections were mounted on chrome-alum/gelatin-coated microscope slides, dehydrated in an ascending ethanol series and coverslipped using Entellan (Merck, Darmstadt, Germany).

To label 5HT immunoreactive neurons, slices were washed in PBS. Unspecific binding sites were blocked for one hour at room temperature using 5% NGS in PBS containing 0.5% TrX. The primary antibody solution was applied for two days at 4°C and consisted of rabbit-anti-5HT antiserum (1:2000, ImmunoStar, Cat No. 20080) 5% NGS, and 0.5% TrX in PBS. After several rinses in PBS containing 0.5% TrX, sections were incubated overnight at 4°C with goat anti-rabbit IgG conjugated to Alexa Fluor 488 (1:200; Molecular Probes, Eugene, OR), 5% NGS and 0.5% TrX in PBS. After rinsing in PBS, sections were mounted on chromalum/gelatin-coated microscope slides, dehydrated in an ascending ethanol series and coverslipped using Entellan (Merck, Darmstadt, Germany).

### Synapsin/phalloidin staining

For general observation of brain structures we combined f-actin labeling using phalloidin with anti-synapsin immunostaining, as described previously by others [52, 53]. Brains were fixed in 4% paraformaldehyde in PBS at 4°C overnight. After washing in PBS, brains were embedded in 5% low-melting point agarose (Typ I-A, low EEO, Sigma-Aldrich Chemie GmbH, Steinheim, Germany) and sectioned in the frontal plane at 100 μm using a vibrating blade microtome (VT1200 S, Leica Microsystems, Wetzlar, Germany). To increase antibody permeability, sections were treated with subsequent rinses in 2% TrX (10 min) and 0.2% TrX (2x10 min) at room temperature. After pre-incubation with 2% NGS, 0.2% TrX in PBS for 1 h at room temperature, sections were incubated for 3 days at 4°C with the primary antibody solution containing anti-synapsin (1:50, SYNORF 1, RRID: AB_2315425), 2% NGS, and 0.2% TrX in PBS. After at least 5 rinses in PBS, the sections were incubated overnight at 4°C with a solution containing goat-anti-mouse antiserum conjugated to Cy3 (1:300, Dianova, Hamburg, Germany), 1% NGS and 0.2 units phalloidin conjugated to AlexaFluor 488 (Life-technologies, Thermo Fischer scientific Inc., Rockford IL, USA) in PBS (500 μl per brain). After five rinses in PBS, sections were transferred to 60% glycerol in PBS for at least 30 min, before they were mounted and coverslipped in 80% glycerol.

### Image acquisition and processing

Fluorescence was detected using a confocal laser scanning microscope (Leica TCS SP5, Leica Microsystems, Wetzlar, Germany). Depending on the required resolution we used either a 10x or 20x oil immersion objective (HC PL APO 10x/0.40 IMM CS, HCX PL APO 20x/0.70 lmm Corr Lbd. bl.) or a 63x glycerin immersion objective (HCX PL APO 63X/1.3 GLY CORR CS 21; all Objectives: Leica). The fluorophores were excited using the following lasers and wavelengths: Cy2/Alexa Fluor 488, argon laser, 488 nm; Texas Red, helium neon laser, 594 nm; Cy5, helium neon laser, 633 nm. Image stacks were acquired at a z-step size of 3 μm (10x objective), 1.5 μm (20x objective) or 0.5 μm (63x objective) and a resolution of 1024x1024 pixels per image. All scans were acquired at 200 Hz scanning frequency and a pinhole size of 1 Airy unit. Specimens containing more than one fluorophore were always scanned sequentially.

All primary image processing on the data stacks was carried out using Amira 5.3.3 (FEI Visualization Sciences Group, Mérignac Cedex, France; RRID:nif-0000–00262). For 3D reconstruction of neuropils, data stacks were resampled to a voxel size of 3x3x3 μm^3^. Images were manually segmented based on background staining (medulla, lobula) or tracer injection (MEDRA) in selected slices of all three cardinal planes using the segmentation editor. The outline of the entire neuropil was then interpolated using the wrapping function with each one single subsequential run of the shrinking and the smoothing function.

The morphology of tracer injected neurons was visualized by intensity-based direct volume rendering using the voltex function. To remove background fluorescence, neuronal staining was segmented in each slice using a combination of threshold based and manual selection in the segmentation editor. The resulting labelfield was used to remove any background using the arithmetic function.

In one preparation from an injection experiment (TuLAL1 neurons) image brightness and contrast were locally adjusted using Photoshop (Adobe Systems, San Jose, CA, USA) to visualize weakly stained projections in the lateral bulb.

If not explicitly stated otherwise, confocal images of double labeling experiments show a single confocal section.

### Axes and naming conventions

All positional information is given with respect to the body axis, not the neuraxis. We followed the naming conventions for neuropils suggested by Ito et al. [54] wherever possible. This includes the designation “upper unit” rather than “major unit” (as suggested by Mota et al. [55]) for the large subcompartment of the anterior optic tubercle. To facilitate comparison with other species we coined the new term “lower unit complex” (abbreviated AOTU-LUC) embracing all small neuropils of the anterior optic tubercle (lateral unit and ventrolateral unit in honeybees, 55), as they seem to be functionally connected. For further details see Discussion. The names of neuron types were adapted from publications describing homologous neurons in other insect species (bumblebee: [56]; locust: [57, 58]; monarch butterfly: [59]).

## Results

### Dorsal rim area of the medulla

**Fig 1:**
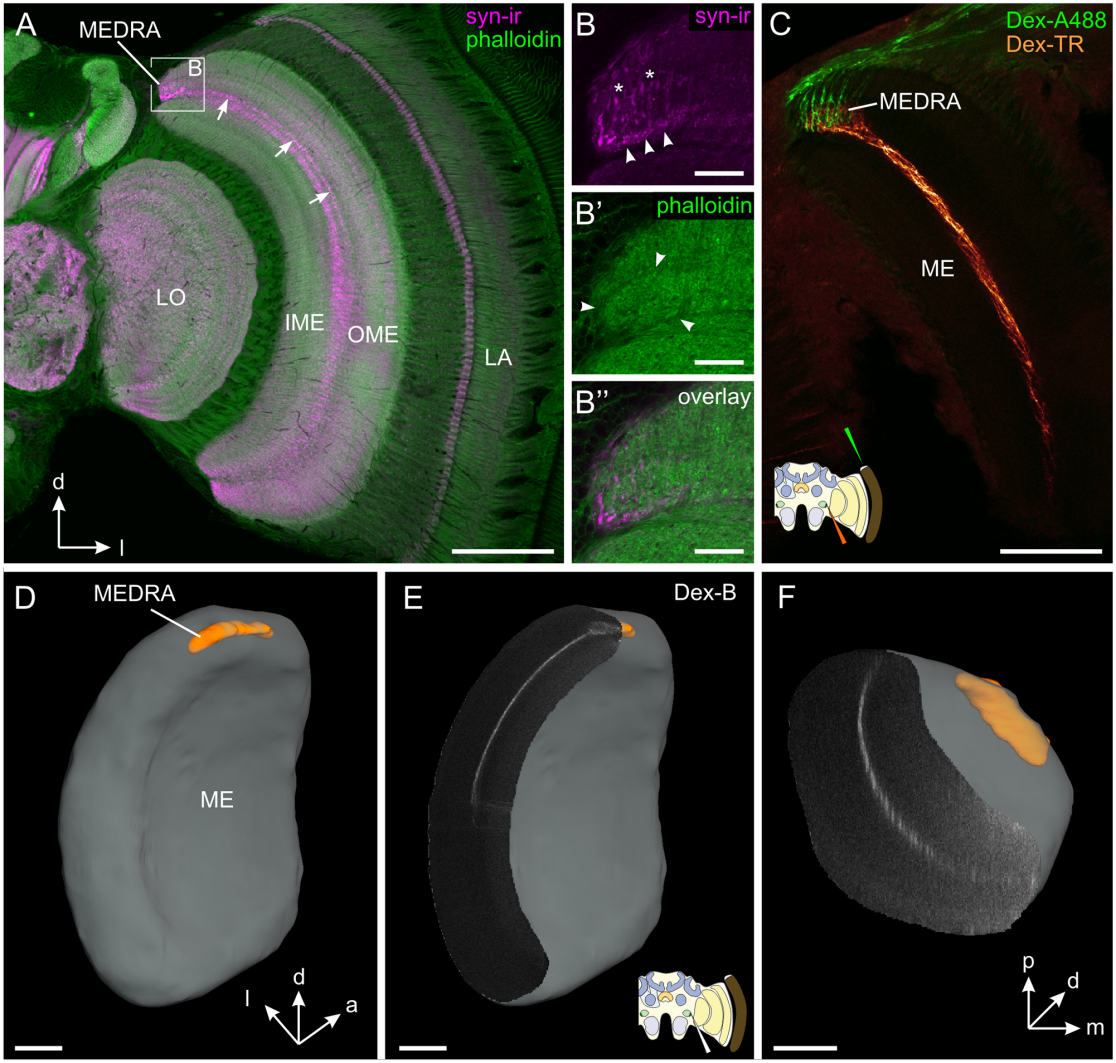
Input layer of the honeybee sky compass system: dorsal rim area of the medulla (MEDRA). (A) Confocal image of anti synapsin (syn-ir, magenta) and phalloidin (green) labeled frontal section of the honeybee optic lobe. Arrows mark a distinct layer within the dorsal half of the outer medulla (OME). (B) Maximum intensity projection (20 slices, z-pitch: 0.5 μm) of the MEDRA shows strongly synapsin-ir labeled terminals, probably of long visual fibers, entering the MEDRA at its dorsal edge (asterisks). Additionally a granular band of unknown origin shows strong synapsin immunoreactivity (arrowheads). (B’) Phalloidin labeling in the dorsal medulla shows a clear delineation of the MEDRA by a darker border (arrowheads). (B”) Overlay of B and B’. (C) Double injection of dextran-Alexa488 (Dex-A488, green) into the dorsal rim area of the compound eye and dextran Texas Red (Dex-TR, orange) into the AOTU-LUC shows common projection area of DRA-photoreceptor terminals and transmedulla neurons of the anterior optic tubercle in the MEDRA. (D-E) 3D-reconstruction of the medulla (gray) and the MEDRA (orange) based on whole mount background staining and dextran Texas Red injection into the AOTU-LUC. (D) Posterior-median view. The MEDRA is an elongated structure at the dorsal posterior edge of the medulla. (E) Frontal section through medulla shows a thin layer that is defined by transmedulla neurons branching out in the MEDRA. Neurons were stained by injection of biotinylated dextran (Dex-B, gray) and labeling with streptavidin-cy3. (F) Ventral view of the same preparation as in D shows that the layer of transmedulla neurons extends almost from the anterior to the posterior end of the medulla. IME, inner medulla; LA, lamina; LO, lobula. Cartoons illustrate injection site. Scale bars: 200 μm in A; 30 μm in B; 100 μm in C-F.

**Fig 2:**
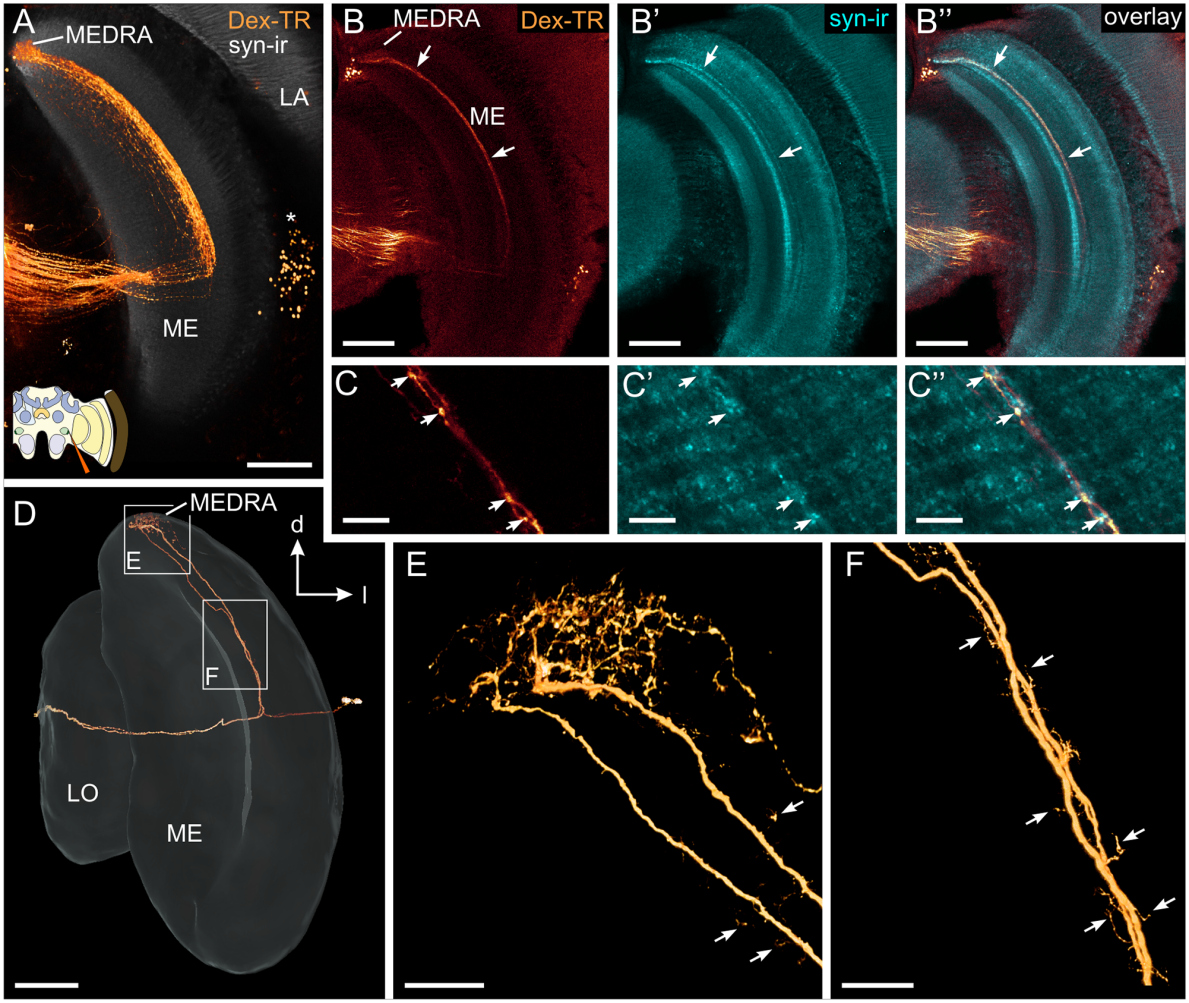
Transmedulla neurons: Ramifications within the medulla. Tracer injection into the lower unit of the anterior optic tubercle labels transmedulla neurons within a thin layer of the dorsal half of the medulla (ME) which extend into the dorsal rim area of the medulla (MEDRA). (A) Direct volume rendering of dextran Texas Red labeling superimposed on synapsin-ir slice. The neurons have their cell bodies at the distal face of the medulla (asterisk), branch in the MEDRA, run through a thin layer within the medulla and enter the 2nd optic chiasm. (B) Transmedulla neurons, labeled through Dex-TR injection into the AOTU-LUC, lie in the same layer (arrows) as a narrow band of synapsin-ir (cyan). (C) Higher magnification/resolution shows that synapsin-ir punctae are next to, but not identical to swellings of the transmedulla neurons, suggesting synaptic input onto the latter. (D-F) Direct volume rendering of two neurobiotin-injected sibling transmedulla neurons. (D) Extracellular iontophoretic injection of Neurobiotin into the AOTU-LUC labeled with streptavidin-Cy3 shows two sibling transmedulla. From an extensive meshwork of branches within the MEDRA, a single, unbranched neurite runs in dorsoventral direction through the medulla. (E, F) Higher magnification/resolution images of MEDRA (E) and a more ventral part of the neurite running dorsoventrally through the medulla (F). Arrows indicate small processes both medially and laterally of the neurite. LA, lamina; LO, Lobula. All views in frontal plane. Scale bars: 100 μm in A, B, and D; 20 μm in C, E, and F.

Using an antibody against the synaptic vesicle protein synapsin (syn-ir) and phalloidin, which binds to filamentous actin, we identified the dorsal rim area of the medulla (MEDRA) as a small neuropil area, at the dorsal edge of the medulla (Fig 1). It was not spatially separated from the medulla proper, but rather integrated into the outer medulla (Fig 1A). Syn-ir strongly labeled two distinct structures within the MEDRA. Most conspicuously, numerous processes entered the neuropil from dorsal (asterisks in Fig 1B) and terminated in large irregular swellings which were most likely the terminals of long visual fibers. At the ventral face of the MEDRA syn-ir labeled a band of granular appearance (arrowheads in Fig 1B). In the phalloidin staining the MEDRA was delineated by a darker outline (arrowheads in Fig 1B’). Tracer application to the dorsal rim area of the compound eye (DRA) showed that long visual fibers terminate exclusively in the MEDRA (Fig 1C). Additional injection of a different tracer into the lower unit complex of the anterior optic tubercle (AOTU-LUC) revealed overlapping branching areas of DRA photoreceptors and transmedulla neurons throughout the MEDRA (Fig 1C). Based on the ramifications of these transmedulla neurons we created a 3D reconstruction of the MEDRA. In addition, the medulla proper was reconstructed based on background staining. The 3D reconstruction shows the MEDRA as an elongated structure which was located posteriorly in the dorsal medulla (Fig 1D–1F). Transmedulla neurons branching in the MEDRA extended ventrally approximately half way to two thirds in the dorsoventral axis (Fig 1E, see also Fig 2A, 2B and 2D), but were present almost throughout the entire anterior-posterior axis (Fig 1F). Double labeling of syn-ir and transmedulla neurons stained through tracer injection into the AOTU-LUC, revealed that a narrow layer, highlighted by syn-ir, corresponded to the layer in which the transmedulla neurons ran (Figs 1A and 2B–2B” arrows). Closer inspection showed that the syn-ir was in close proximity to the transmedulla neurons, but did not colocalize with them suggesting synaptic output of yet unknown neurons onto the transmedulla neurons (Fig 2C–2C”).

The cell bodies of the transmedulla neurons were located between the outer face of the medulla and the inner face of the lamina (Fig 2A, asterisk). The primary neurite entered the medulla and gave off two branches approximately in the middle of the neuropil. A presumably axonal branch projected through the 2^nd^ optic chiasm and the lobula to the AOTU-LUC. The other, presumably dendritic, neurite ran along the innermost layer of the outer medulla to the MEDRA, where it gave rise to an extensive field of arborizations. For a closer morphological investigation of this type of neuron, we used extracellular iontophoretic dye injections into the AOTU-LUC, which allowed us to stain small numbers of transmedulla neurons (Fig 2D–2F). The extensive ramifications within the MEDRA of only two individual neurons suggest input from numerous photoreceptors of the DRA (Fig 2E) onto each neuron within the population. Higher resolution imaging of the neurites showed studding with spine-like appendages that extended both laterally and medially suggesting input not only in the MEDRA but along the entire dorsoventral extent of the neurite (arrows Fig 2E and 2F).

### Anterior optic tubercle

**Fig 3:**
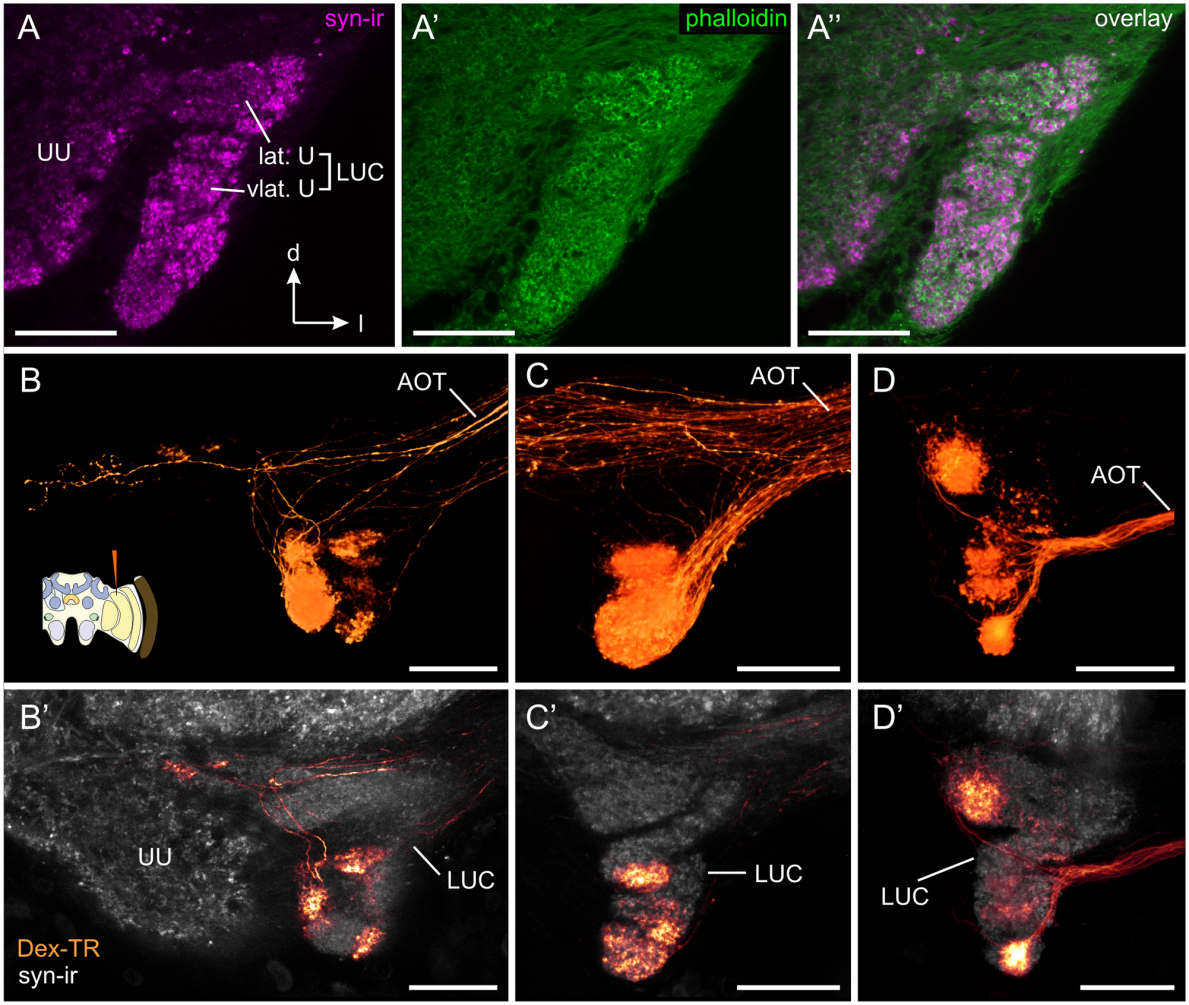
Transmedulla neurons: Ramifications within the lower unit complex of the anterior optic tubercle (AOTU-LUC). (A) Confocal image of synapsin-ir (syn-ir, magenta) and f-actin labeling (phalloidin, green) of the anterior optic tubercle. The AOTU-LUC has been previously divided into the lateral unit (lat. U) and the ventrolateral unit (vlat. U) [55]. Synapsin/phalloidin labeling reveals that these two units are further structured into a complicated assembly of multiple small subcompartments. B-D) Direct volume rendering of AOTU-LUC projections from transmedulla neurons labeled through dextran Texas Red (Dex-TR) injection into the MEDRA. In each sample a different combination of focal projection areas is stained. Cartoon in (B) illustrates injection site. B’-D’) Single confocal sections (B’, C’) and maximum intensity projection of 10 adjacent slices (D’) of the neurons shown in B-D, combined with anti-synapsin labeling (grey). Projections from MEDRA neurons are exclusively found in the AOTU-LUC. AOT, anterior optic tract; UU, upper unit of the anterior optic tubercle. All views in frontal plane. All scale bars: 30 μm.

To identify the target neuropil of the transmedulla neurons, we injected dextran Texas Red into the MEDRA. Central projections were exclusively found in the AOTU. We therefore investigated the AOTU using syn-ir/phalloidin labeling to get a better understanding of its anatomical fine structure. As shown previously by Mota et al. [55] the AOTU-LUC is not a continuous neuropil, but is further segmented into a lateral unit and a ventrolateral unit (Fig 3A). Beyond these two main compartments, syn-ir/phalloidin labeling revealed further segmentation into a complicated aggregation of numerous small subregions with irregular shape (Fig 3A–3A”). Tracer injection into the MEDRA further confirmed this observation: each subcompartment was innervated by a different fascicle given off by the anterior optic tract (Fig 3B–3D). In each individual preparation, we found staining in a different set of subcompartments that were usually in close spatial proximity to each other, suggesting a spatial mapping between the medulla and the subcompartments of the AOTU-LUC. A large, highly stained area around the injection site, however, precluded the systematic investigation of such a spatial mapping.

**Fig 4:**
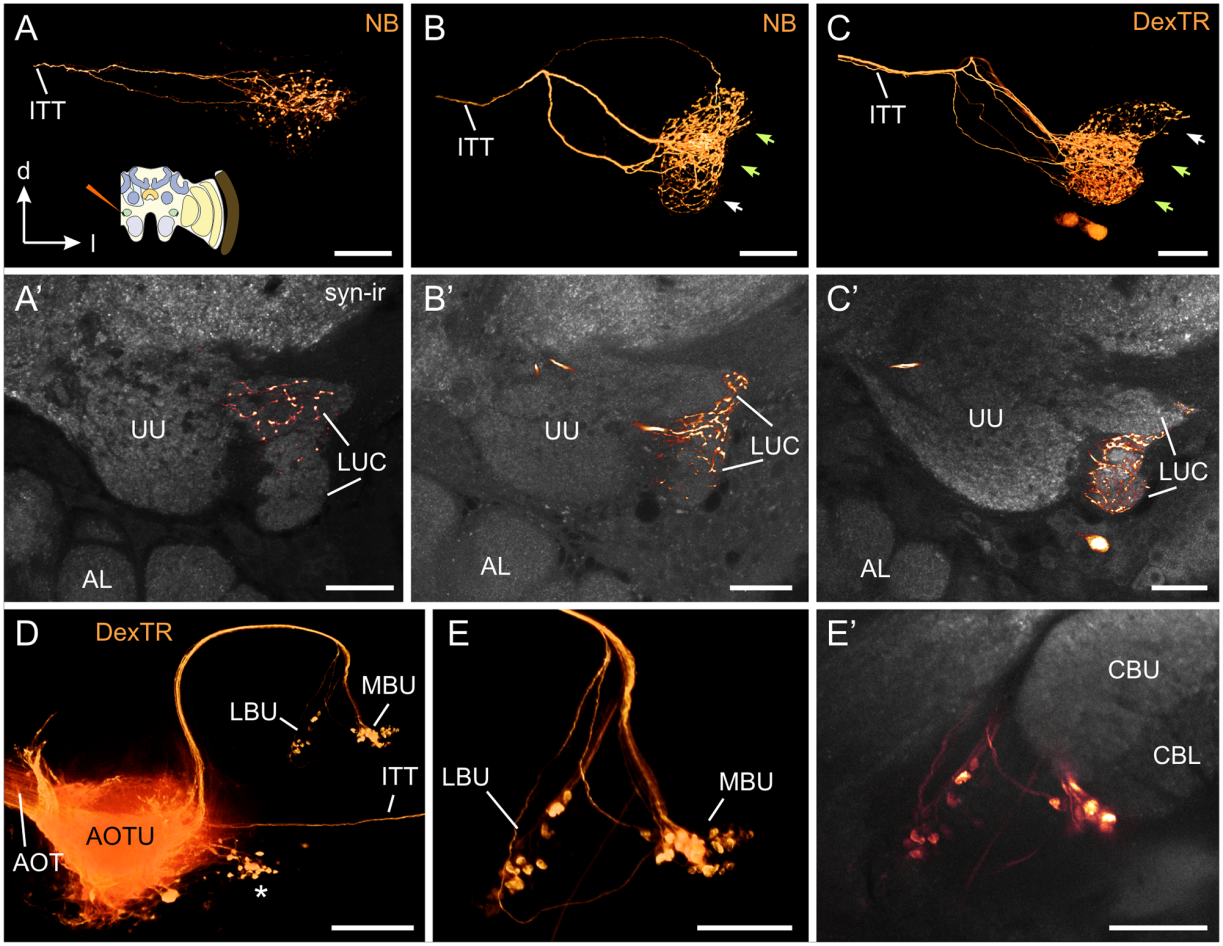
Central projections of neurons in the lower unit complex of the anterior optic tubercle (AOTU-LUC). (A-E) Neurobiotin (NB) injection into the AOTU-LUC reveals central projection areas. Cartoon in A illustrates injection site. (A-C) Direct volume rendering from projections in the contralateral AOTU-LUC shows three morphologically different types of TuTu1 neuron (TuTu1a, TuTu1b, TuTu1c). (A’-C’) Maximum intensity projections of six (A’) or three (B’, C’) adjacent slices from preparations shown in A-C, combined with anti-synapsin labeling (syn-ir, grey). (A’) TuTu1a neurons connecting only the dorsal areas of the AOTU-LUC. (B’, C’) Two similar types of TuTu1 neuron with ramifications in the ventral and median areas of the AOTU-LUC. These neurons had dense (green arrows) and sparse (white arrows) ramification areas. While TuTu1b neurons ramified densely within their dorsal, and sparsely within their ventral branching areas (B’), the opposite was true for TuTu1c neurons (C’). (D, E) Projections of TuLAL1 neurons from the AOTU-LUC to the medial and lateral bulb. (D) Overview (direct volume rendering) shows course of the axons which run within the AOTU-LAL tract. The tract separates into two fascicles that innervate the lateral or the medial bulb (LBU, MBU), respectively. The cell bodies of TuLAL1 neurons were located medially of the AOTU (asterisk). Also stained are axons of TuTu1 neurons that run in the intertubercle tract (ITT). (E) Direct volume rendering of large synaptic terminals of TuLAL1 neurons within the median and the lateral bulbs. (E’) Maximum intensity projection of three adjacent slices of preparation shown in E combined with anti-synapsin labeling (grey) illustrates the projection areas of TuLAL1 neurons with respect to the central complex. AL, antennal lobe; CBU, central body upper division; CBL, central body lower division; UU, upper unit of AOTU. All views in frontal plane. Scale bars: 30 μm in A-C; 100 μm in D; 50 μm in E.

Compartmentalization of the AOTU-LUC was also observed when injecting dye directly into this neuropil and observing the projections within the complementary neuropil on the contralateral side. We were able to identify three morphological types of heterolateral interneuron interconnecting the AOTU-LUC of both sides through the intertubercle tract (Fig 4A–4C). Following the naming conventions in other insects this type of neuron was termed tubercle-tubercle neuron 1 (TuTu1) [57–59]. The first subtype (TuTu1a, Fig 4A and 4A’) entered the AOTU with its main neurite between the dorsal and ventral lobe of the upper unit and then branched almost exclusively in the dorsalmost compartment of the AOTU-LUC (termed lateral unit by Mota et al. [55]). The second and the third subtype (TuTu1b, Fig 4B and 4B’ TuTu1c, Fig 4C and 4C’) also entered the AOTU from median, but their neurites first bent dorsally and subsequently deflected downwards where they gave rise to several large sidebranches that then innervated several subcompartments within the ventral two thirds of the AOTU-LUC (collectively termed ventrolateral unit by Mota et al. [55]). While the ramifications of these two subtypes generally overlapped, the dorsal field of ramifications was dense in TuTu1b neurons (Fig 4B, green arrows) and sparse in TuTu1c neurons (Fig 4C, white arrow), whereas the opposite was true for the ventral ramification area. Both types seemed to spare the neuropil area that was innervated by TuTu1a neurons. In all three types of neuron the distribution of processes within the AOTU-LUC appeared similar in the ipsilateral and contralateral brain hemisphere (S1 Fig).

### Central projections

Most successful dye injections into the AOTU-LUC stained neurons that projected through the AOTU-LAL tract around the vertical lobe of the mushroom body. As the tract deflected downwards the fibers segregated into a medial and a lateral fascicle projecting to two focal areas in the vicinity of the central complex termed the lateral and medial bulb (Fig 4D and 4E). According to the naming conventions in desert locusts these neurons were termed TuLAL1a if they projected to the lateral bulb and TuLAL1b, if they projected to the medial bulb [57, 60]. The presynaptic terminals of TuLAL1 neurons were conspicuously large having a diameter of up to 8 μm. To check whether the projections within the medial and the lateral bulb were associated with specific compartments of the AOTU-LUC, we analyzed the subtypes of TuTu1 and TuLAL1 neurons that were stained in the same brains. Most injections that predominantly labeled projections in the lateral bulb also stained TuTu1a neurons in the contralateral AOTU-LUC, suggesting that the dorsalmost compartment of the AOTU-LUC (lateral unit) is connected to the lateral bulb. Likewise injections labeling projections in the medial bulb usually also stained TuTu1b and/or TuTu1c neurons, suggesting a link between the ventral compartments of the AOTU-LUC (ventrolateral unit) and the medial bulb.

### Transmedulla neurons are in close proximity to PDF-ir, 5HT-ir and GABA-ir fibers

**Fig 5:**
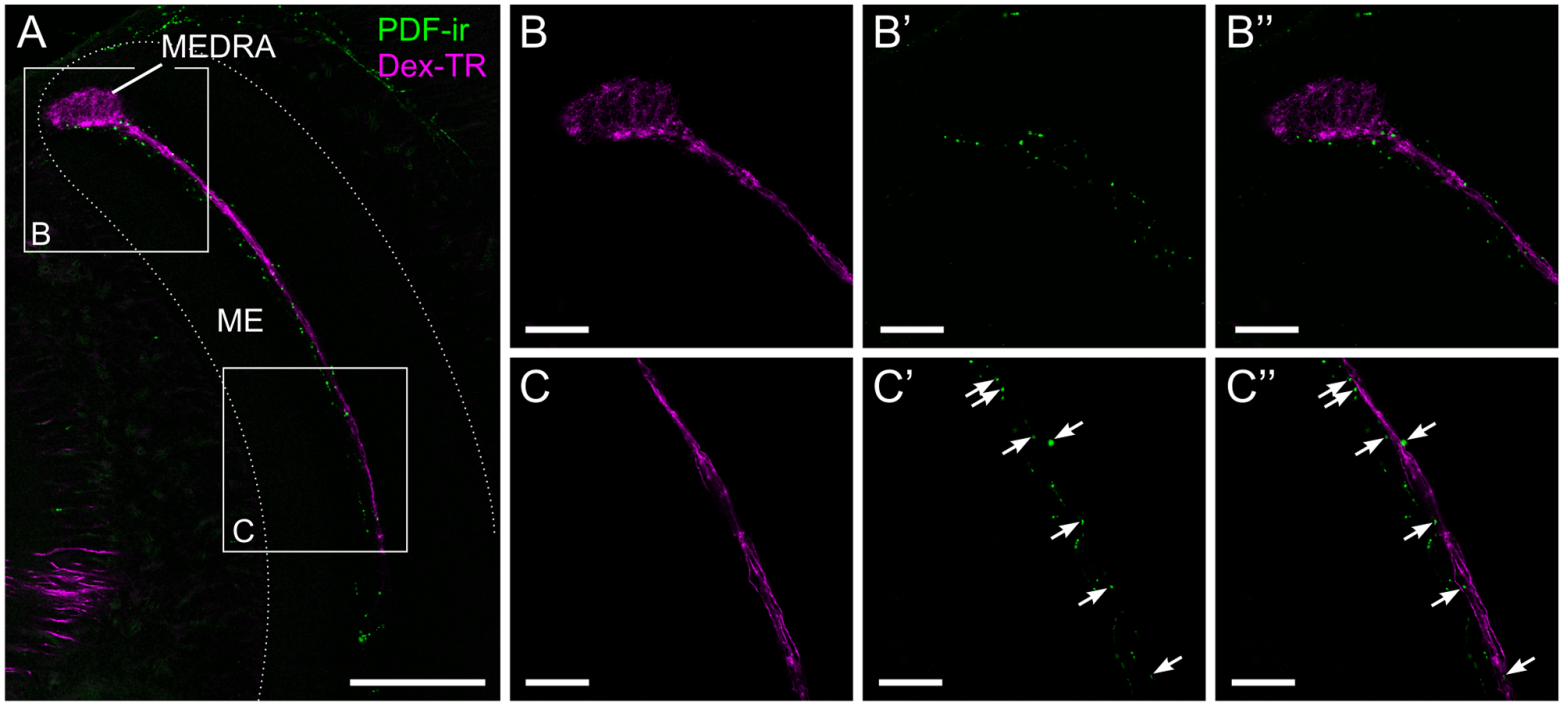
Spatial relationship between transmedulla neurons and PDF-ir fibers. (A) Confocal image of PDF-immunoreactive neurons (PDF-ir, green) and transmedulla neurons stained by dextran Texas Red injection into the AOTU-LUC (DEX-TR, magenta). Both types of neuron run in the same layer of the medulla (ME). (B, C). Higher magnification/resolution images of the areas indicated in A shows close proximity, but no colocalization of the two stainings. (B) PDF-ir is not found within the dorsal rim area of the medulla (MEDRA), only at its ventral edge. (C) PDF-ir sparsely labels small punctae which are in close proximity to the transmedulla neurons. All views in frontal plane. Scale bars: 100 μm in A; 30 μm in B, C.

The neuropeptide pigment dispersing factor (PDF) has been shown to be an output signal of the insect circadian clock [20–22]. To locate possible sites of interaction between the circadian clock and the sky compass network, and thus potential neural substrates for time-compensation, we used an antiserum directed against the PDF of honeybees. Spatial overlap between PDF-immunoreactivity (PDF-ir) and ramifications of sky compass neurons was exclusively found in the medulla (Fig 5). PDF-ir fibers sparsely innervated a narrow layer in the medulla from the dorsal to the ventral edge of the neuropil. The PDF-ir neurons in the medulla strongly overlapped with the layer defined by the transmedulla neurons of the sky compass system (Fig 5A). Imaging at high magnification/resolution showed that the two fiber systems did not colocalize. Rather we observed small PDF-ir punctae in close proximity to transmedulla neurons of the sky compass system, allowing for the possibility of PDF release onto these neurons. Overlap was not found within the MEDRA (Fig 5B) but only along the vertical passage of the transmedulla neuron’s fibers through the medulla (Fig 5C). Experiments where an antiserum against crustacean β-pigment dispersing hormone (PDH) was used instead of the *Apis* PDF antiserum gave virtually identical staining patterns in the medulla (S2 Fig).

**Fig 6:**
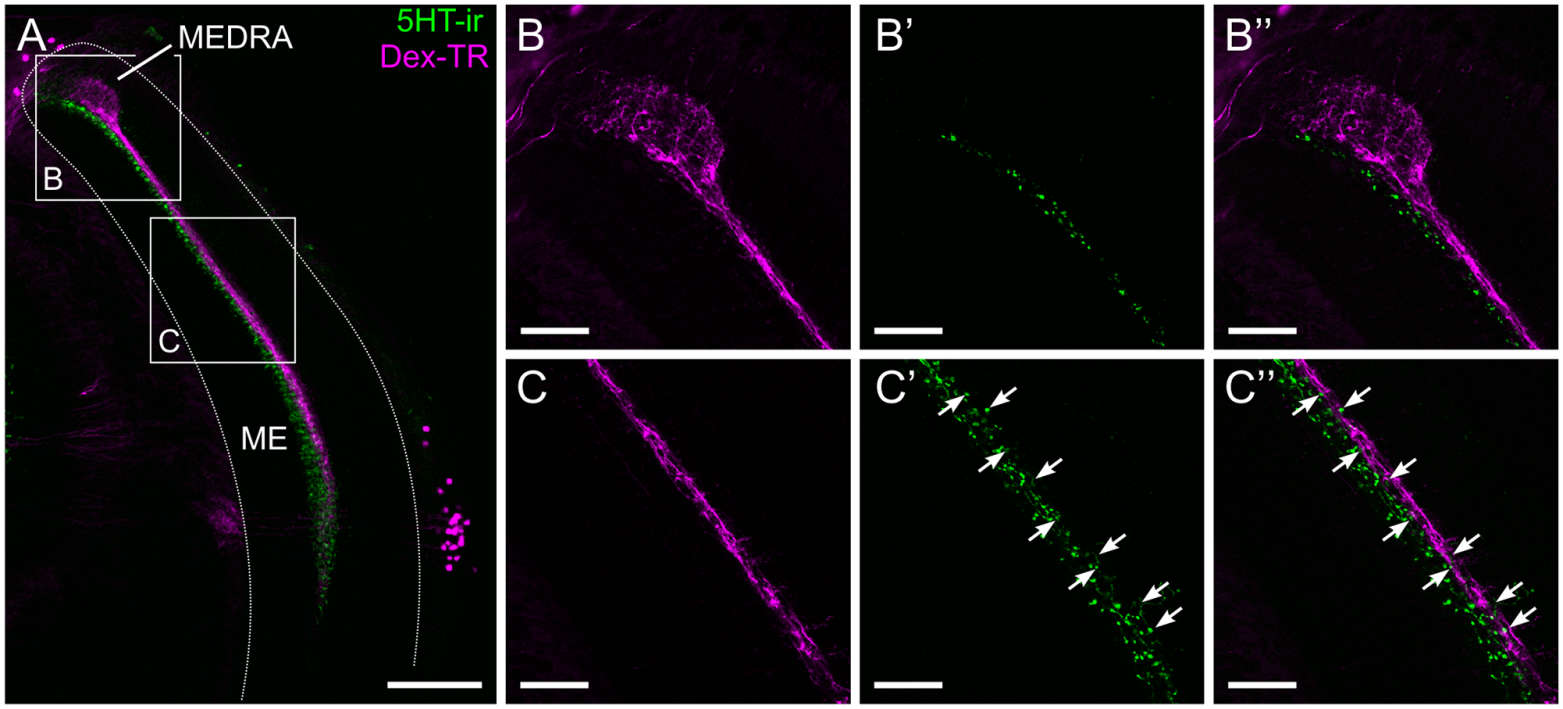
Spatial relationship between transmedulla neurons and 5HT-ir fibers. (A) Confocal image of 5HT-immunoreactive (5HT-ir) neurons (green) and transmedulla neurons labelled by dextran biotin/streptavidin Texas Red (magenta). Both stainings are restricted to the dorsal and medial parts of the medulla. (B, C) Higher magnification/resolution images of the areas indicated in A show close proximity, but no colocalization of the two stainings. (B) The dorsal rim area of the medulla (MEDRA) is devoid of 5HT-ir. (C) Transmedulla neurons overlap with the band of 5HT-ir, but the latter is wider and extends more medially. ME, medulla. All views in frontal plane. Scale bars: 100 μm in A; 30 μm in B, C.

It has previously been shown that 5HT immunoreactivity (5HT-ir) is only found in the dorsal half of one of the median layers of the medulla [42]. 5HT has also been shown to mediate several circadian effects within the insect visual system. To test whether 5HT-ir neurons also overlap with neurons of the sky compass system in the honeybee’s medulla, we combined 5HT-immunostaining with tracer injection into the AOTU-LUC (Fig 6). We found a clear overlap between the immunostaining and transmedulla neurons of the sky compass network projecting to the AOTU-LUC (Fig 6A). The antiserum against 5HT stained a broader layer of dense punctae overlapping the layer of transmedulla neurons on its lateral side. A striking feature of the 5HT-ir in relation to the transmedulla neurons was, that both sets of neuron only occupied approximately the dorsal half to two thirds of the neuropil. As with the PDF-ir we observed no overlap within the MEDRA (Fig 6B) but only along the vertical passage through the medulla (Fig 6C) Again, no colocalization was detected, i.e. injection and immunostaining labelled two distinct neuron populations, allowing for the possibility of 5HT release onto neurons of the sky compass system.

**Fig 7:**
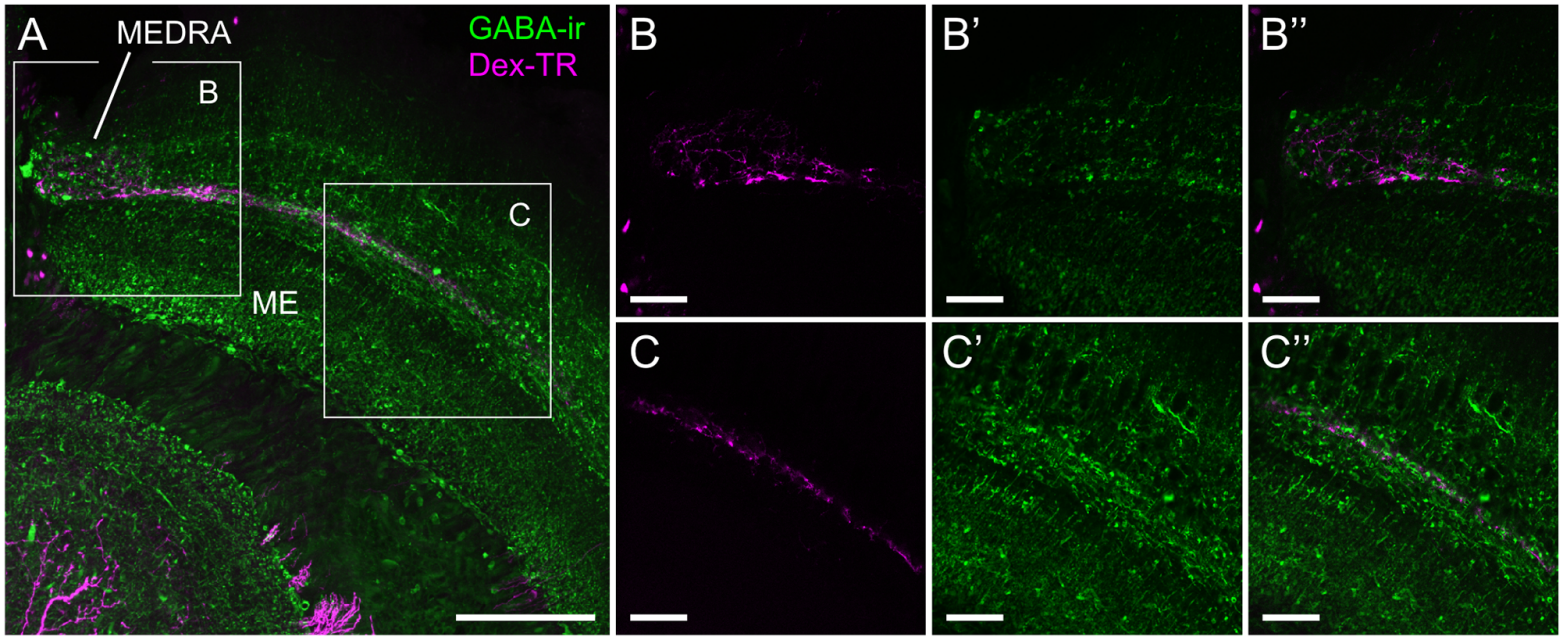
Spatial relationship between transmedulla neurons and GABA-ir fibers. (A) Confocal image of GABA-ir neurons (green) and transmedulla neurons stained by dextran Texas Red injection into the AOTU-LUC (magenta). GABA-ir is found throughout the medulla, but is more concentrated in some layers than in others. (B, C) Higher magnification/resolution images of the areas indicated in A show close proximity, but no colocalization of the two stainings. (B) GABA-ir is also present within the dorsal rim area of the medulla (MEDRA). (C) Varicose GABA-ir is in close proximity to the transmedulla neurons. ME, medulla. All views in frontal plane. Scale bars: 100 μm in A; 30 μm in B, C.

In search of further candidate substances that could provide modulatory input to the sky compass system we combined immunostaining using an antiserum against γ-aminobutyric acid (GABA) with tracer injection into the AOTU-LUC (Fig 7). Dense GABA immunoreactivity (GABA-ir) was found throughout the medulla as previously described by Schäfer and Bicker [61], including the layer defined by the transmedulla neurons of the sky compass system (Fig 7A). At high resolution/magnification, we identified GABA-ir of beaded appearance in close proximity to the transmedulla neurons. Different from PDF-ir and 5HT-ir, GABA-ir was found along the entire length of the transmedulla neurons, including the MEDRA. No colocalization was found between the two stainings, suggesting that they represent different neuron populations.

### The AOTU-LUC is devoid of PDF-ir, 5HT-ir and GABA-ir

**Fig 8:**
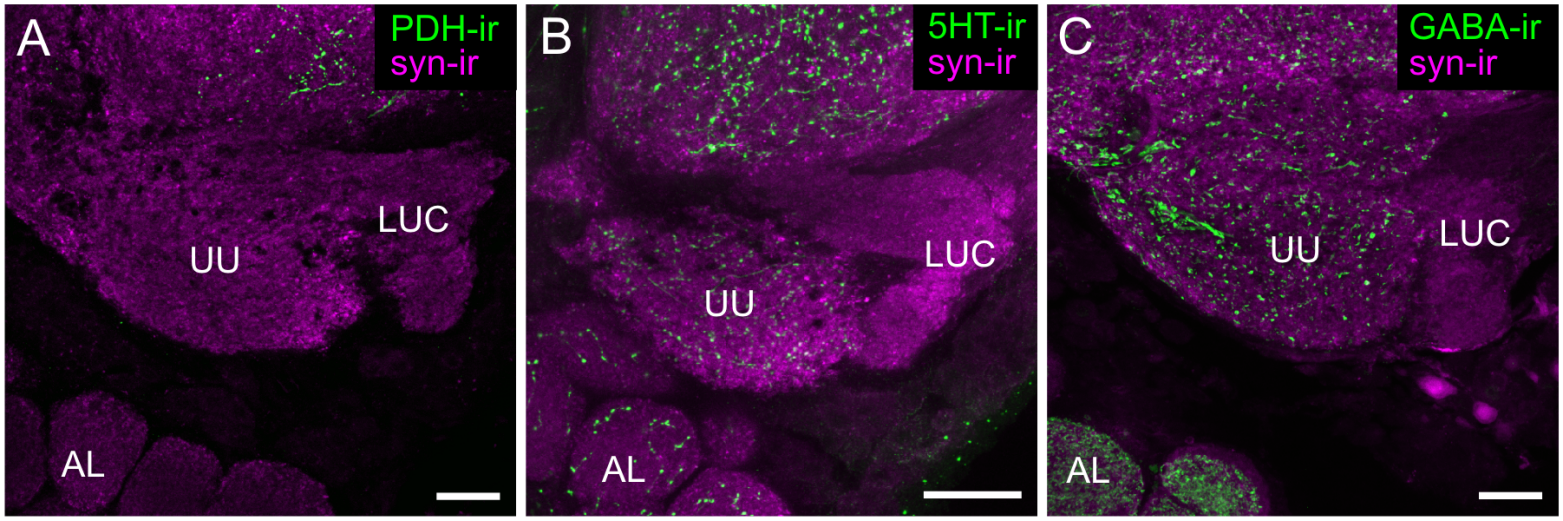
Absence of PDF-, 5HT, and GABA-ir in the lower unit complex of the anterior optic tubercle (AOTU-LUC). Synapsin-immunoreactivity (syn-ir, magenta) and immunoreactivity to antisera against PDH (PDH-ir), 5HT (5HT-ir) and GABA (GABA-ir), shown in green, in the anterior optic tubercle. (A) The entire AOTU is devoid of PDH-ir. (B) 5HT-ir was found in the upper unit of the anterior optic tubercle (UU), but not in the AOTU-LUC. (C) GABA-ir, green was found in the AOTU-UU, but not in the AOTU-LUC. AL, antennal lobe. All views in frontal plane. All scale bars: 30 μm.

To assess whether there are other potential modulation sites within the honeybee sky compass pathway, we studied immunoreactivity against PDH, 5HT and GABA within the AOTU. While the upper unit of the AOTU, which is not part of the sky compass pathway, was labelled by antisera against 5HT and GABA, the AOTU- LUC was devoid of immunoreactivity to all three antisera (Fig 8A–8C).

## Discussion

**Fig 9:**
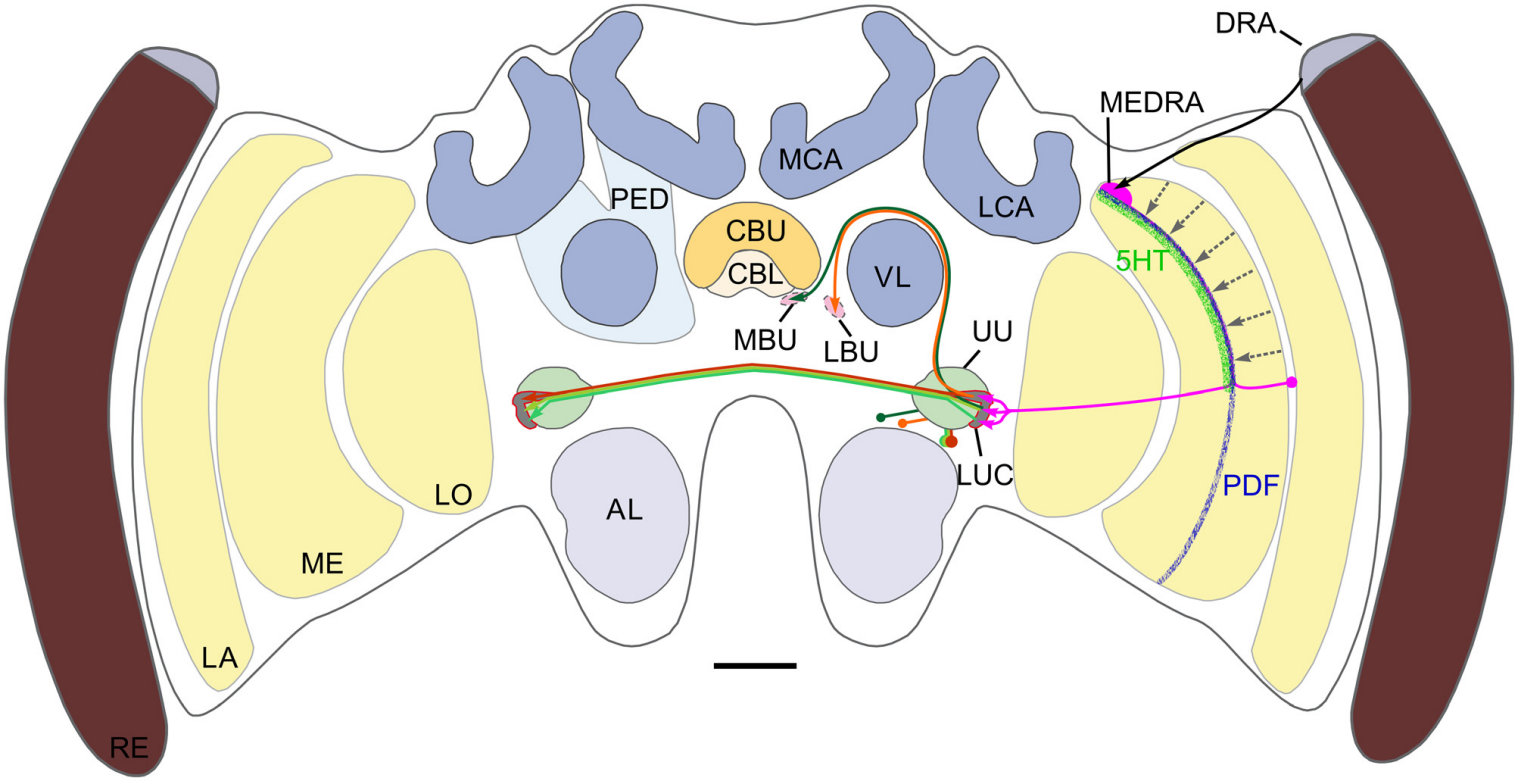
Summary of the main findings. Frontal schematic diagram of the honeybee brain illustrates the main neuropils and the sky compass pathway. The dorsal rim area of the eye (DRA) is connected to the dorsal rim area of the medulla (MEDRA) through long visual fibers. Transmedulla neurons project from the MEDRA to the lower unit complex of the anterior optic tubercle (LUC). TuLAL1 neurons project from the LUC around the vertical lobe of the mushroom body (VL) to the median and lateral bulb (MBU, LBU). Three Types of TuTu1 neurons project to the contralateral LUC. Stippled grey lines in medulla (ME) indicate hypothetical unpolarized light input pathways. Also shown is immunoreactivity in the medulla to antisera against pigment dispersing factor (PDF) and 5HT (5HT). AL, antennal lobe; CBL, lower division of the central body; CBU, upper division of the central body; LA, lamina; LCA and MCA, lateral and medial calyx of the mushroom body; LO, lobula; PED, pedunculus. Scale bar: 200 μm.

In this study we combined anatomical tracing techniques with immunocytochemistry against PDF, 5HT and GABA, to unravel the sky compass pathway of the honeybee and to find potential sites of circadian and neuromodulatory input to this system. We show a neuronal pathway that originates in the dorsal rim area of the medulla, connects to the AOTU-LUC and projects to the bulbs near the central complex (Fig 9). One major finding was that transmedulla neurons connecting the MEDRA to the AOTU-LUC have numerous short spines along their passage through the medulla. This suggests that they receive additional input, like unpolarized light information and/or neuromodulatory input there. PDF-ir, 5HT-ir and GABA-ir neurons all branch in the vicinity of these neurons in the medulla pointing towards modulatory input, some of it potentially of circadian nature.

### Comparison to the sky compass pathway of other insect species

The sky compass pathway has previously been described in locusts [57, 62], bumblebees [56] and desert ants [63]. The DRA visual fibers and some elements from the AOTU have also been reported from the honeybee [55, 64] and the monarch butterfly [12, 59, 65]. With the exception of LoTu1 neurons, which bilaterally connect the lobulae and AOTUs of both brain hemispheres in locusts [66], we found counterparts for all previously described types of sky compass pathway neurons in the honeybee. As expected from the close relationship of honeybees and bumblebees the systems in the two species were extremely similar. The similarities between the locust and the honeybee sky compass pathway were also striking, particularly considering the evolutionary distance between holometabolous and hemimetabolous insects.

One conspicuous difference, however, was the dorsoventral extent of the transmedulla neurons. While in honeybees, as described before in bumblebees [56], these neurons extended from the MEDRA only about halfway ventrally, they span the entire dorsoventral extent of the medulla in locusts [15, 57]. This probably creates a receptive field covering only the area above the horizon in bees, while in locusts it is to be expected that the receptive field extends ventrally. The functional implications of this finding are not clear at present.

### The anterior optic tubercle

The anterior optic tubercle of insects is composed of one large and up to three small subunits. (locust: [57]; blowfly: [67]; sphinx moth: [68]; honeybee: [55]; butterflies: [69, 70]; bumblebee: [56]). In most insects the large compartment is called the upper unit (it has previously been called the major unit in honeybees [55]) and is probably homologous across species. It has been implicated in the processing of color and motion in honeybees [55, 71, 72]) and in figure-ground discrimination and phototaxis in *Drosophila* [73, 74]. The smaller units have been assigned varying names and are not easily homologized across species. However, in all species that have been investigated so far, the upper unit is not part of the sky compass pathway, whereas all the small compartments are (locust: [58]; monarch butterfly: [12, 59]; bumblebee: [56]; honeybee: this study). Heinze et al. [59] demonstrated a spatial mapping between the small compartments of the AOTU and the projection areas of TuLAL1 neurons in the bulb (of which monarch butterflies only have one per hemisphere). We found a similar mapping in the honeybee, where TuLAL1 neurons from different subcompartments of the AOTU-LUC either projected to the lateral or the medial bulb, suggesting two separate input pathways into the central complex. Although in locusts there is only one small subunit, called the lower unit, there are also two sets of TuLAL1 neurons projecting to the lateral and the medial bulb in the lateral complex [58, 60]. Taken together the findings from different species suggest that the small units in holometabolous insects have been derived from a single small unit as represented by the AOTU-LU in the locust brain. To facilitate comparison between species, we suggest using the term lower unit (AOTU-LU) in species that possess only a single small AOTU subunit, and the term lower unit complex (AOTU-LUC) in species with multiple small subunits, to collectively signify all of them.

### Integration of polarized and unpolarized sky compass cues

It is well known that polarization-sensitive neurons in the sky compass system of insects also code for the azimuth of unpolarized light spots (locust: [10, 11]; monarch butterfly: [12]; dung beetle: [13]), but the integration site of these different stimuli is unknown. While integration could theoretically take place in the AOTU itself, the morphology of the transmedulla neurons studied here and their position within the pathway strongly suggest that these are the neuronal elements performing the integration. The overlap of their prominent ramifications in the MEDRA with terminals of DRA photoreceptors suggests polarized light information input in this neuropil. Recent data from the locust show that the MEDRA is organized in a retinotopical way [75]. Therefore, their extensive dendritic fields in the MEDRA indicate that these neurons sample polarization information from a large area of the sky. This agrees with electrophysiological data from a different type of polarization-sensitive Neuron (POL1) in the medulla of crickets which receives input from the entire DRA [76]. Large-field integration is an important property of neuronal systems processing celestial polarized light, because it makes the system robust against local irregularities of the polarization pattern [76–78]. In contrast, accurate detection of the solar azimuth requires the opposite: narrow receptive fields.

The transmedulla neurons studied here are likely to fulfill this condition as well. The spines along their dorsoventral passage through the medulla suggest additional input to this segment of the neurons. The insect visual system is organized retinotopically, i.e. neighboring points of the environment are represented by neighboring neuronal elements [79]. According to the anatomy of the transmedulla neurons one can therefore assume a receptive field in the shape of a narrow vertical bar directed at a certain azimuth. Thus, each neuron in the population could be tuned to respond maximally when the sun is located at a certain azimuth with respect to the animal and due to their long extent in dorsoventral direction, this response should be independent of solar elevation.

While the DRA photoreceptors seem to project directly onto the transmedulla neurons, the unpolarized light information is probably neither provided directly by long visual fibers nor by lamina monopolar cells, because these elements terminate in the outer layers (layers 1 and 2) of the medulla [80]. Instead it has to be expected that a connection is made by local interneurons within the medulla, as indicated in Fig 9 (grey stippled arrows).

### Circadian input into the sky-compass system

While multimodal integration can help to make systems more robust by adding redundancy, it bears the risk that different input channels provide conflicting information. In the case of the sky compass system, this problem can occur through changes in the spatial relationship between non-zenithal *E*-vectors and the solar azimuth, arising from changes in solar elevation [11]. In neurons of the locust AOTU, two physiological properties help to avoid cue conflicts. First, an area of about 50° around the sun, where these changes are particularly prominent, provides no polarization information to the system due to its subthreshold degree of polarization [81]. Second, these neurons continuously adjust their tuning throughout the day so that changes in the angle between *E*-vectors in the sky and the solar azimuth are compensated for [11]. Similar properties have been shown for neurons in the monarch butterfly [82]. Clearly, such a compensation mechanism has to be implemented upstream of, or directly at, the integration site of polarization-information and solar azimuth information. Furthermore it is necessary that either only the polarization channel or only the unpolarized light channel is modulated (or at least that they are modulated differentially). Both conditions could be met through the interaction of transmedulla neurons and the circadian system in the honeybee. PDF-ir fibers were found in close proximity to transmedulla neurons as they pass through the medulla, while the MEDRA was devoid of PDF-ir. PDF is an output signal of the insect circadian clock and has been shown to cause dose- and daytime-dependent changes (increases and decreases) in the sensitivity of visual interneurons in the medulla of crickets [26].

Similarly to PDF-ir, 5HT-ir spared the MEDRA, but otherwise overlapped prominently with the transmedulla neurons of the sky compass pathway. In the optic lobe of crickets, 5HT levels undergo circadian fluctuations [25]. Furthermore 5HT has been shown to downregulate the sensitivity of visual interneurons in the medulla of crickets and the lobula of honeybees [26, 29]. It is therefore conceivable that selective local release of PDF and 5HT onto the transmedulla neurons of the sky compass pathway aids to shape the solar azimuth tuning of neurons further downstream in a circadian manner.

Our findings fit well with a previous study in monarch butterflies that also located a potential connection between the sky-compass system and the circadian clock in a central layer of the medulla [65]. These authors used an antiserum against CRYPTOCHROME-1 (CRY1), which is part of the molecular clock network, as a proxy for neurons of the circadian system. Similar to PDF-ir fibers in the honeybee, CRY1-ir fibers in the monarch had fine varicose branchings restricted to a central layer of the medulla. Terminals of DRA photoreceptors, traced by injection of fluorescent dyes terminated in the dorsal part of this medulla layer. This was interpreted as an indication of circadian input into the sky compass system.

In locusts, PDH-ir fibers only branch in the outermost layers of the medulla and hence do not overlap with the transmedulla neurons in this species [35]. This is a profound species specific difference between bees and locusts and particularly interesting regarding the fact that compensation for changes in the angle between the solar azimuth and celestial *E*-vectors has been shown in the AOTU of locusts, i.e. downstream of the transmedulla neurons [11]. It raises the question how time information is integrated in the medulla of these animals. El Jundi et al. showed that several types of polarization- and azimuth sensitive large-field tangential neurons branching throughout the same layer as the transmedulla neurons have additional branches in the accessory medulla [15]. These neurons could act as the interface between the circadian clock and the peripheral sky-compass network in locusts.

In two studies on locusts and monarch butterflies the distribution of preferred *E*-vector angles from all recorded neurons shows a substantially larger scatter than the corresponding distribution of preferred azimuth tuning angles from the same cells [11, 12]. This was interpreted as an indication that *E*-vector tuning rather than azimuth tuning is adjusted in the course of the day to compensate for diurnal changes arising from changes in solar azimuth. Such a mechanism would call for modulatory input into the DRA rather than the unpolarized input sites of the transmedulla neurons as reported here. Since there is no physiological description of the bee’s sky-compass system to date, it is not clear if a compensation for changes in solar elevation in bees is carried out in these insects as it is in locusts and monarch butterflies.

Circadian input into the sky compass system could also have other functions as well. Bees indeed compensate for changes in solar azimuth and this specifically requires adjustment of the azimuth sensitivity somewhere in the network. It is also conceivable that a peripheral circadian modulation of the sky compass network simply globally modulates sensitivity to account for different light levels. Physiological studies are needed in the future to distinguish between these possibilities.

### GABA-ir in the medulla

GABA-ir is abundant in the optic lobe of a variety of insect species (honeybee: [61]; hawkmoth: [83]; locust: [84]; swallowtail butterfly: [85]). GABA usually has inhibitory effects on the postsynaptic neurons, but its specific functions in the optic lobe of honeybees are unknown. In flies, GABA plays a crucial role in shaping the tuning of visual interneurons in the lobula plate. In direction sensitive movement detectors, called H1, selective blocking of GABA_A_ receptors using picrotoxin reverses directional selectivity [86]. Similarly, the preference for small object over large field motion in FD1 neurons is inverted by picrotoxin [87]. In the sky compass pathway of honeybees GABA might also play a role in shaping the tuning of the neurons. Unlike PDF-ir and 5HT-ir, GABA-ir was present throughout the MEDRA, as reported previously for locusts [62], and could therefore interact with the processing of both polarized and unpolarized-light information. GABA can act both neuromodulatory or as a regular transmitter. The GABA-ir fibers in proximity to the transmedulla neurons could therefore provide photic inputs into these fibers rather than modulating them. In the MEDRA, however one can assume that the principal input comes from the photoreceptors of the DRA and that the GABA-ir fibers serve a different function.

## Conclusion

Our results show that the sky compass pathway is highly conserved between different insect species. Immunocytochemistry combined with tracer injection suggests interaction between PDF-ir, 5HT-ir and GABA-ir fibers with transmedulla neurons. Our data are a first hint of circadian and neuromodulatory input to the sky compass pathway in the medulla of honeybees. Future experiments, including electron microscopy and especially neurophysiological experiments combined with pharmacology, will help to better understand the function of the sky compass system and its modulation.

## Supporting Information

S1 Fig Comparison of ipsi-and contralateral ramifications of three TuTu1 neuron types. (A, B) Ipsi- (A, ipsi) and contralateral (B, contra) ramifications of a single TuTu1a neuron stained by extracellular iontophoretic dye injection of Neurobiotin (NB, orange). Neuropil stained through synapsin immunoreactivity (syn-ir, gray). Maximum intensity projections showing entire AOTU (A, 117 slices, z-pitch: 0.5 μm) and ramifications in the lower unit complex of the anterior optic tubercle only (LUC; A’, ipsilateral, 16 slices; B, contralateral, 6 slices, z-pitch: 0.5 μm). Ramifications are restricted to the dorsalmost compartments of the (LUC) on both sides of the brain. On the ipsilateral side, TuLAL1a neurons and some unidentified neuron types are stained as well. B is identical to Fig 4A’. (C, D) Ipsi- (C) and contralateral (D) ramifications of a single TuTu1b neuron stained by extracellular iontophoretic dye injection of NB. Maximum intensity projections showing entire AOTU (C, 42 slices, z-pitch: 3 μm) and ramifications in the LUC only (C’, ipsilateral, 15 slices; D, contralateral, 30 slices, z-pitch: 0.5 μm). On both sides of the brain, TuTu1b neurons have characteristic ramification areas with only sparse innervation of the ventralmost compartments of the LUC (arrows in C’, D) close to the cellular cortex (asterisk) and a dorsally tapered denser ramification area in the medial LUC compartments. Also stained on the ipsilateral side are TuLAL1 neurons and some unidentified neuron types. (E, F) Contra- (E) and ipsilateral ramification areas (F) of TuTu1c neurons stained through dextran Texas-Red injection (Dex-TR) in two different preparations. Maximum intensity projections showing entire AOTU (E, 145 slices, z-pitch: 0.5 μm) and ramifications in the LUC (E’, contralateral, 21 slices; D, ipsilateral, 3 slices, z-pitch: 0.5 μm). This type of neuron has branches predominantly in the ventral LUC, including the ventralmost area that is only sparsely innervated in TuTu1b neurons. Also stained is an axon of a heterolateral lobula neuron running in the anterior optic tract (arrowhead). F is identical to Fig 4C’. AL, antennal lobe; ITT, intertubercle tract; UU upper unit of anterior optic tubercle; d dorsal; l, lateral; m, medial; Scale bars: 30 μm.
(TIF)

S2 Fig Spatial relationship between transmedulla neurons and PDH-ir fibers. (A) Confocal image of PDH-immunoreactive neurons (PDH-ir, green) and transmedulla neurons stained by dextran Texas Red injection into the AOTU-LUC (DEX-TR, magenta) combined with immunostaining against synapsin (gray). Both types of neuron run in the same layer of the medulla (ME). (B, C). Higher magnification/resolution images of approximate areas indicated in A shows close proximity, but no colocalization of the two stainings. (B) PDH-ir is not found within the dorsal rim area of the medulla (MEDRA), only at its ventral edge. (C) PDH-ir sparsely labels small punctae which are in close proximity to the transmedulla neurons. All views in frontal plane. Scale bars: 100 μm in A; 30 μm in B, C.
(TIF)
