# Supplementary figures and images for: Transmedulla Neurons in the Sky Compass Network of the Honeybee (Apis mellifera) Are a Possible Site of Circadian Input
Source: PLoS One. 2015 Dec 2;10(12):e0143244. doi: 10.1371/journal.pone.0143244 (PMC4667876; doi:10.1371/journal.pone.0143244)

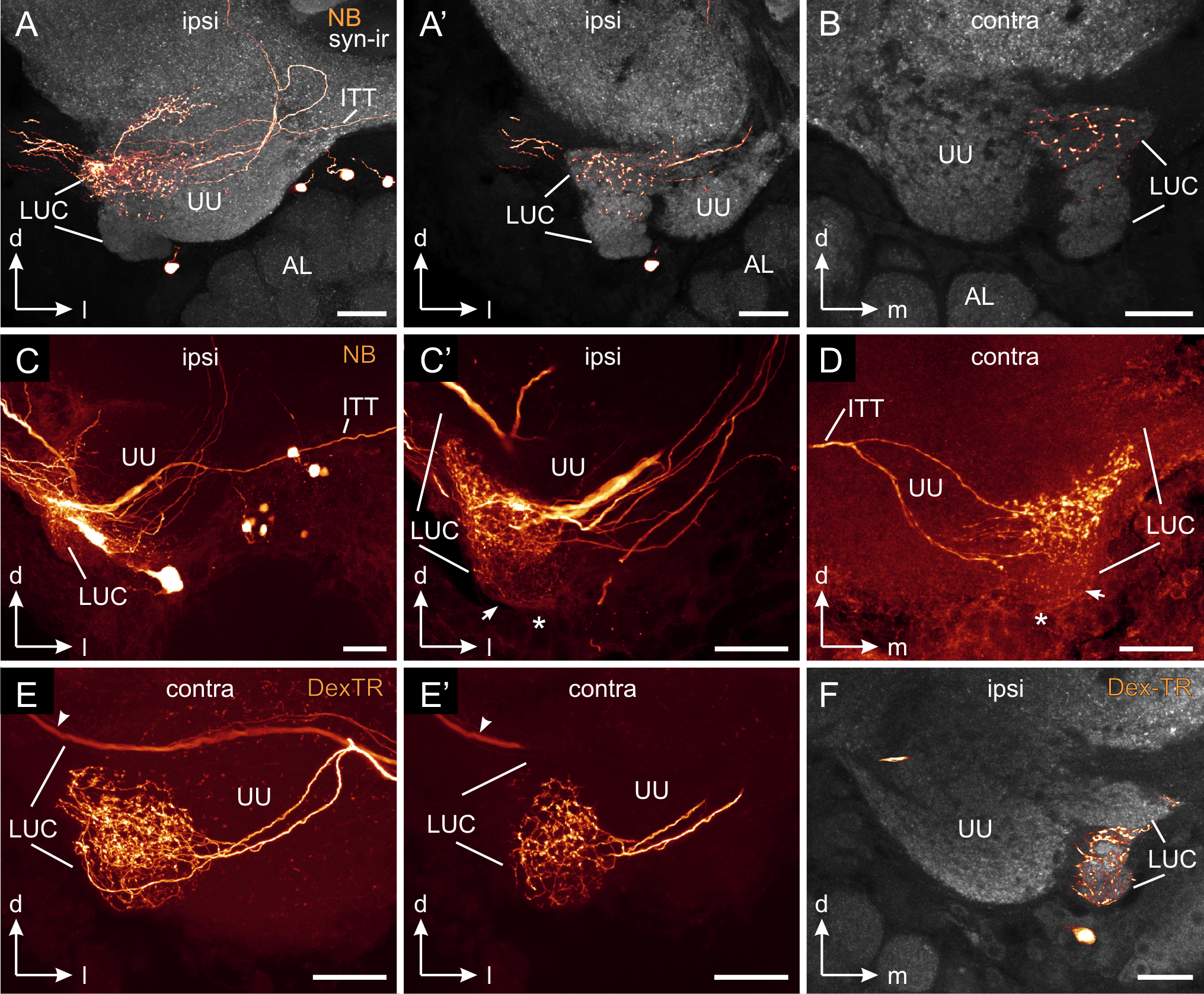

Supplement: S1 Fig — (A, B) Ipsi- (A, ipsi) and contralateral (B, contra) ramifications of a single TuTu1a neuron stained by extracellular iontophoretic dye injection of Neurobiotin (NB, orange). Neuropil stained through synapsin immunoreactivity (syn-ir, gray). Maximum intensity projections showing entire AOTU (A, 117 slices, z-pitch: 0.5 μm) and ramifications in the lower unit complex of the anterior optic tubercle only (LUC; A’, ipsilateral, 16 slices; B, contralateral, 6 slices, z-pitch: 0.5 μm). Ramifications are restricted to the dorsalmost compartments of the (LUC) on both sides of the brain. On the ipsilateral side, TuLAL1a neurons and some unidentified neuron types are stained as well. B is identical to Fig 4A’. (C, D) Ipsi- (C) and contralateral (D) ramifications of a single TuTu1b neuron stained by extracellular iontophoretic dye injection of NB. Maximum intensity projections showing entire AOTU (C, 42 slices, z-pitch: 3 μm) and ramifications in the LUC only (C’, ipsilateral, 15 slices; D, contralateral, 30 slices, z-pitch: 0.5 μm). On both sides of the brain, TuTu1b neurons have characteristic ramification areas with only sparse innervation of the ventralmost compartments of the LUC (arrows in C’, D) close to the cellular cortex (asterisk) and a dorsally tapered denser ramification area in the medial LUC compartments. Also stained on the ipsilateral side are TuLAL1 neurons and some unidentified neuron types. (E, F) Contra- (E) and ipsilateral ramification areas (F) of TuTu1c neurons stained through dextran Texas-Red injection (Dex-TR) in two different preparations. Maximum intensity projections showing entire AOTU (E, 145 slices, z-pitch: 0.5 μm) and ramifications in the LUC (E’, contralateral, 21 slices; D, ipsilateral, 3 slices, z-pitch: 0.5 μm). This type of neuron has branches predominantly in the ventral LUC, including the ventralmost area that is only sparsely innervated in TuTu1b neurons. Also stained is an axon of a heterolateral lobula neuron running in the anterior [file pone.0143244.s001.tif]

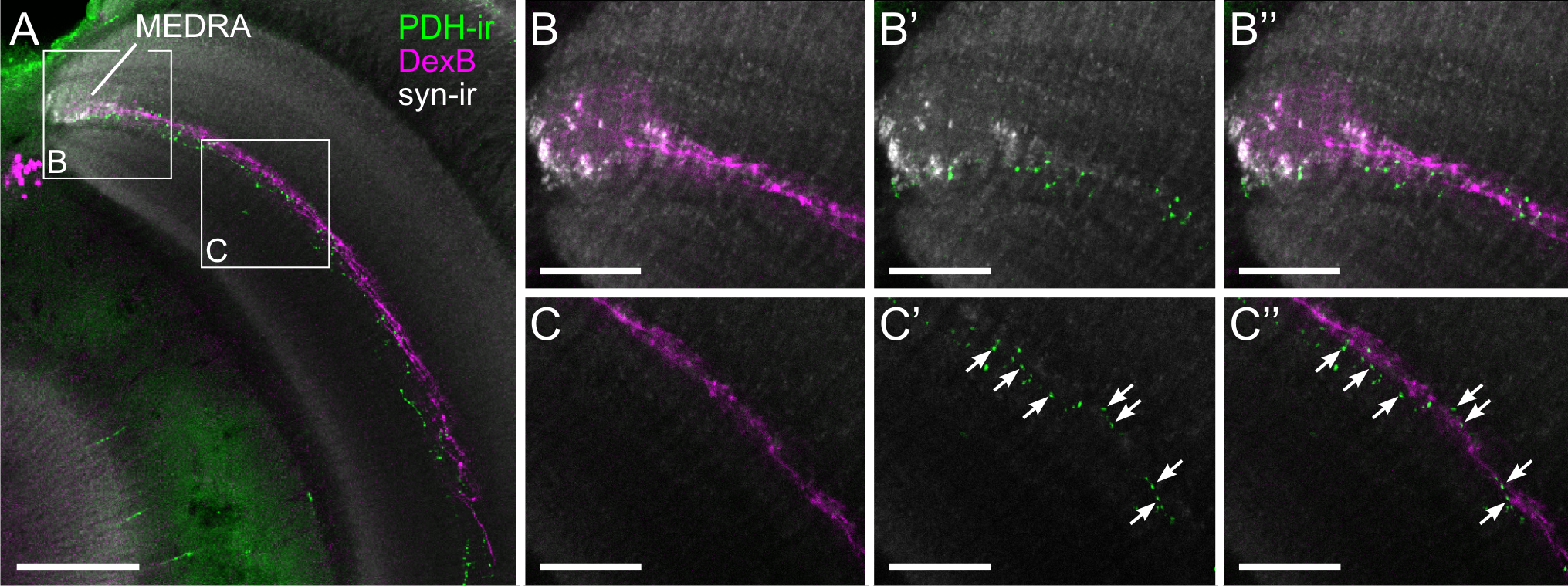

Supplement: S2 Fig — (A) Confocal image of PDH-immunoreactive neurons (PDH-ir, green) and transmedulla neurons stained by dextran Texas Red injection into the AOTU-LUC (DEX-TR, magenta) combined with immunostaining against synapsin (gray). Both types of neuron run in the same layer of the medulla (ME). (B, C). Higher magnification/resolution images of approximate areas indicated in A shows close proximity, but no colocalization of the two stainings. (B) PDH-ir is not found within the dorsal rim area of the medulla (MEDRA), only at its ventral edge. (C) PDH-ir sparsely labels small punctae which are in close proximity to the transmedulla neurons. All views in frontal plane. Scale bars: 100 μm in A; 30 μm in B, C. (TIF) [file pone.0143244.s002.tif]
